# Differential roles of astrocyte and microglia in supporting oligodendrocyte development and myelination in vitro

**DOI:** 10.1002/brb3.152

**Published:** 2013-07-09

**Authors:** Yi Pang, Lir-Wan Fan, Lu-Tai Tien, XueMei Dai, Baoying Zheng, Zhengwei Cai, Rick C S Lin, Abhay Bhatt

**Affiliations:** 1 Department of Pediatrics, University of Mississippi Medical CenterJackson, Mississippi, 39216; 2 School of Medicine, Fu Jen Catholic UniversityXinzhuang Dist, New Taipei City, 24205, Taiwan; 3 Department of Chemistry, Jackson State UniversityJackson, Mississippi, 39217; 4 Department of Pathology, University of Mississippi Medical CenterJackson, Mississippi, 39216; 5 Department of Neurobiology and Anatomical Sciences, University of Mississippi Medical CenterJackson, Mississippi, 39216

**Keywords:** Cytokine, differentiation, glia, in vitro, proliferation

## Abstract

Oligodendrocyte (OL) development relies on many extracellular cues, most of which are secreted cytokines from neighboring neural cells. Although it is generally accepted that both astrocytes and microglia are beneficial for OL development, there is a lack of understanding regarding whether astrocytes and microglia play similar or distinct roles. The current study examined the effects of astrocytes and microglia on OL developmental phenotypes including cell survival, proliferation, differentiation, and myelination in vitro. Our data reveal that, although both astrocytes- and microglia-conditioned medium (ACDM and MCDM, respectively) protect OL progenitor cells (OPCs) against growth factor withdrawal-induced apoptosis, ACDM is significantly more effective than MCDM in supporting long-term OL survival. In contrast, MCDM preferentially promotes OL differentiation and myelination. These differential effects of ACDM and MCDM on OL development are highlighted by distinct pattern of cytokine/growth factors in the conditioned medium, which correlates with differentially activated intracellular signaling pathways in OPCs upon exposure to the conditioned medium.

## Introduction

Traditionally, both astrocytes and microglia have been thought to act as supportive cells in the central nervous system (CNS). It is now widely appreciated that astrocytes and microglia are not only involved in virtually every aspects of neural function in the mature brain (Fields and Stevens-Graham 2002) but also play important roles during CNS development. For example, both astrocytes and microglia are involved in neuronal differentiation, migration, programmed cell death, neurite growth, axon guidance, as well as synaptic formation (Deverman and Patterson 2009). On the other hand, oligodendroglia or oligodendrocyte (OL) appears to resemble more closely to neuron rather than to astrocyte or microglia, in terms of developmental programs and susceptibility to injury. For instance, OL progenitor cells (OPCs) and neurons arise from similar regions of the neuroepithelium, and their fate determination is driven by similar transcription factors (Bradl and Lassmann 2010). Both OPCs and neurons are born in excess, but their numbers are reduced dramatically through programmed cell death (Barres et al.^1992^; Buss et al. 2006). As for cell survival, certain neurotrophic factors are critically involved for both types of cells, while most of those factors are known to be secreted by astrocytes and microglia (Althaus et al. 2008). In addition to these similarities in developmental features, both neuron and OLs are susceptible to certain insults including glutamate excitotoxicity, inflammatory cytokines, and reactive oxidative stress (Leviton and Gressens 2007; Volpe et al. 2008; Volpe 2009).

At present, OL lineage development is well characterized, especially in rodents (Miller 2002; Emery 2010a). Initially, OPCs proliferate in regions where they are generated from multipotent neural progenitor cells, and then migrate to their destination. To ensure that the number of OPCs matches with their corresponding axons, excessive OPCs are eliminated through apoptosis, and the survived cells then undergo terminal differentiation and start to myelinate axons. One of the important features of OL development is that it is largely controlled by extracellular cytokines, most of which are known to be secreted by astrocytes and microglia. Although extensive studies have been conducted to understand the role of individual cytokine on OL development, it is most likely that OLs are exposed to multiple cytokines/growth factors in vivo, thus their biological responses depend on the types of the final signaling pathways activated. However, this is very difficult to assess in vivo. The conditioned medium from astrocytes or microglia offers some aspects of the in vivo environment and may provide information to better elucidate the roles of astrocytes and microglia during OL development.

Generally it is thought that astrocytes and microglia play important roles in OL development; however, such view is largely based on studies of individual cytokines/growth factors which are known to be secreted by astrocytes and/or microglia (Deverman and Patterson 2009). Based on these lines of information, it is very conceivable that astrocytes and microglia may play a differential role for OL developmental phenotypes. With regard to myelination, a limited studies suggest that astrocytes may enhance myelination process (Ishibashi et al. 2006; Watkins et al. 2008), whereas the role of microglia is largely unknown. Therefore, the aim of this study was to address the above questions by examining OL development and myelination in cell cultures upon exposure to astrocyte- and microglia-conditioned medium (ACDM and MCDM), which contains many secreted cytokines all together.

## Material and Methods

### Chemicals

Dulbecco's modified Eagle medium (DMEM)/Ham's F12 and F15 medium, fetal bovine serum (FBS), neural basal medium (NBM), B27 supplements, 7.5% bovine serum albumin (BSA), platelet-derived growth factor (PDGF-AA), basic fibroblast growth factor (bFGF), penicillin/streptomycin, and 2.5% trypsin were purchased from Invitrogen (Carlsbad, CA). The antibodies against NG2, O4, Rip, adenomatosis polyposis coli (APC), myelin basic protein (MBP), glial fibrillary acid protein (GFAP), CD-11b, phosphorylated neurofilament (pNF), and antibodies against the phosphorylated as well as total form of Akt, extracellular signal-regulated protein kinase1/2 (Erk1/2), cAMP response element binding protein (CREB), signal transducer and activator of transcription 3 (STAT3), were from Millipore (Billerica, MA). The XTT (2,3-Bis-(2-methoxy-4-nitro-5-sulfophenyl)-2H-tetrazolium-5-carboxanilide) assay kit and BrdU (5-bromo-2'-deoxyuridine) kit were purchased from Roche (Indianapolis, IN). TUNEL (terminal deoxynucleotidyl transferase dUTP nick end labeling) staining kits were purchased from Serologicals (Norcross, GA). Neutralizing antibodies for PDGFaa, bFGF and insulin-like growth factor-1(IGF-1), and enzyme-linked immunosorbent assay (ELISA) kits for ciliary neurotrophic factor (CNTF) and IGF-1 were from R and D systems (Minneapolis, MN). The antibody array kit was from Raybiotech (Norcross, GA). The advanced electrochemiluminescence (ECL) detection kits were purchased from GE Healthcare Biosciences (Piscatway, NJ).

### Primary cell cultures

#### OL, astrocyte, and microglia cultures

Primary cultures of OPCs, astrocytes, and microglia were prepared from neonatal rat cortices at postnatal day 1 (P1), as described previously (Pang et al. 2000, 2010). Briefly, the cortices of P1 rat brain were separated from the surrounding tissue, trypsinized, and maintained in DMEM/F12 with 10% FBS. Upon reaching confluency, the flasks were shaken on an orbit shaker at 180 rpm for 2 h. The floating cells were filtered through a cell strainer (40 μm pore size), centrifuged, and plated in a noncoated flask. After 10 min, the flasks were gently shaken by hand to dislocate loosely attached cells, which were then discarded. The firmly attached microglia were then trypsinized and seeded on poly-l-lysine-coated coverslips sitting in 12-well plates. To prepare OPCs, the flasks were shaken again at 200 rpm overnight. The supernatant was transferred to a noncoated Petri dish and incubated at 37°C for 1 h. The Petri dish was gently swirled to release loosely attached cells and the supernatant was transferred into poly-l-lysine-coated flasks. OPCs were maintained in a chemically defined medium (CDM), which contains DMEM/F12, 0.1% BSA, 100 μmol/L putrescine, 20 nmol/L progesterone, 10 nmol/L sodium selenium, 20 nmol/L biotin, 5 μg/mL cysteine, 5 nmol/L hydrocortisone, 5 μmol/L insulin, 50 μmol/L transferrin, 2 nmol/L l-glutamine, and penicillin/streptomycin), supplemented with PDGF-AA and bFGF (final concentration 10 ng/mL, each). To enrich astrocyte population, the remaining cells in the flasks were shaken again for 6 h at 220 rpm, and the supernatant was discarded. Cells remained in the flasks were mostly astrocytes, which were further subcultured with a 1:6 split ratio, and fed with DMEM/20% FBS. When astrocytes reached 80% confluence, they were trypsinized and seeded onto coverslips sitting in 12-well plates at a density of 1 × 10^5^ per well. The purity of glial cultures was assessed by immunocytochemistry using glial specific markers (i.e., NG2 and O4 for OPCs, GFAP for astrocytes, and CD-11b for microglia). Typically, the purity of OPC, astrocyte, and microglia cultures was 95.4%, 97.7%, and 94.2%, respectively (estimated from three separate primary cultures).

#### Neuron-OL myelination coculture

The coculture was prepared from the cortex of embryonic day 16 rat fetuses, as described elsewhere (Pang et al. 2012). Briefly, the cortices were collected in cold Hank's balanced salt solution (HBSS) and chopped into small pieces using a surgical blade. Tissue was digested in trypsin/ethylenediaminetetraacetic acid (EDTA) solution for 10 min at 37°C. The reaction was stopped by addition of trypsin inhibitor-DNase-I solution, and tissue was dissociated into single cell suspension by passing through 1 mL fine pipette tip for five times. Cells were then seeded on poly-l-lysine-coated coverslips at a density of 0.4 × 10^5^ per cm^2^. Cultures were maintained in N2-NBM (1:1) with nerve growth factor (NGF) (50 ng/mL) and neurotrophin-3 (NT-3) (10 ng/mL) for the first 7 days of culture, and then in insulin-free N2-NBM (4:1) afterwards. We have previously shown that extensive myelination could be detected in the cocultures by 4 weeks (Pang et al. 2012).

### Preparation of the conditioned medium and treatment

In order to compare the effects of the two types of glia-conditioned medium on OL phenotypes, the density of astrocytes and microglia as well as the volume of the medium were kept identical. Briefly, purified astrocytes or microglia were seeded on poly-l-lysine-coated coverslips sitting on the bottom of 12-well plates, at a density of 1 × 10^5^ per well in 200 μL medium. Cells were allowed to be attached to the coverslips for 1 h. After washing three times with 1× HBSS, 1 mL of insulin-free CDM was added into each well. Medium added to empty wells was served as the control. The plates were returned to the CO_2_ incubator. After 24 h, the conditioned medium was collected, filtered with a 0.45 μm filter, and stored at −80°C until use.

### Cell survival/death assay

Cell survival/death rate was assessed by two methods. Short-term survival (<72 h) was determined using the XTT assay. OPCs were seeded on poly-l-lysine-coated 96-well plates at a density of 1 × 10^4^ per well. Cell survival rate was calculated as a percentage (%) of the treatment over that of the control, as previously described (Pang et al. 2000). Cell survival/death rate in long-term cultures (>72 h) was determined by counting the number of pyknotic nuclei versus intact nuclei stained with DAPI (4',6-diamidino-2-phenylindole), and the results were represented as a percentage of the number of intact nuclei to total nuclei (intact + pyknotic). We have previously shown that this approach can reliably estimate the long-term OL survival in cultures (Pang et al. 2010).

### Cell proliferation assay

Cell proliferation was assessed by BrdU labeling method. Briefly, OPCs were seeded onto poly-l-lysine-coated coverslips at a density of 1 × 10^4^ per coverslip (2.2 mm diameter). After overnight incubation, the medium was changed without growth factors (PDGF/bFGF) and continued to culture for 24 h. Following washing in HBSS, cells were treated with the conditioned medium or the control for 48 h. Cells treated with PDGF-AA (10 ng/mL) was served as the positive control. BrdU (1 μmol/L) was added to the medium 12 h before being fixed and processed for immunostaining. The number of BrdU+ cells as well as total nuclei (PI counter staining) was counted in 10 randomly selected high power view fields (100×) for each coverslip, three coverslips per condition. Cell proliferation is represented as the percentage of BrdU+ cells to total cells (DAPI-counterstained nuclei). Data were obtained from three independent sets of experiments.

### Immunocytochemistry

Cells were seeded on poly-l-lysine-coated coverslips at a density of 5 × 10^4^ per coverslip. To label mitochondria, 25 nmol/L Mitotracker Red CMXRos (Invitrogen) was added to the cultures 30 min prior to the end of the treatment. Cells were rinsed twice with ice-cold phosphate buffered saline (PBS) and were fixed with 4% paraformaldehyde (PFA) for 15 min at room temperature (RT). Following washing in PBS, cells were permeabilized with 0.1% Triton X-100 and blocked with 5% normal serum/1% BSA and 0.1% Triton X-100 in PBS for 1 h. Cells were then incubated with the Rabbit anti-Bax antibody (1:50) for 2 h, followed by Cy2-conjugated secondary antibody (1:200) and DAPI (10 nmol/L) for 1 h at RT. The coverslips were washed, mounted, and viewed under a Olympus fluorescence microscope (Center valley, PA). Images of cells labeled with Bax immunostaining (green), Mitotracker (red), and DAPI (blue) in the same view field (150×) were captured with a CCD camera, and superimposed using the Adobe Photoshop (version 7.0) software (Adobe Systems Inc., San Jose, CA).

### Assessment of myelination

The myelinating cocultures grown on poly-l-lysine-coated coverslips (sitting on 12-well plates) were treated with conditioned medium starting from DIV7 until DIV35. To avoid significant altering myelinate culture medium, which has been shown to affect myelin formation in the coculture system (Pang et al. 2012), the conditioned medium was concentrated 10-folds using Microcon filters with a 5 kDa pore size (Millipore). The concentrated conditioned medium was then diluted 1:10 into the myelinating medium for treatment. Fresh myelinating medium (with diluted conditioned medium) was changed every 3 days. On DIV35, the myelination cultures were fixed with 1% PFA, permeabilized with 1% triton, and double immunostained with anti-MBP and pNF antibodies. The myelination index was calculated as a ratio of MBP+ to pNF+ area using ImageJ software (NIH, Bethesda, MD), as described previously (Pang et al. 2012). Data were obtained from triplicated coverslips per condition in three independent experiments.

### Antibody array, ELISA, and immunoblotting

To compare patterns of secreted cytokine in the conditioned medium, ACDM and MCDM were blotted against a protein array membrane which can detect 90 cytokines. The procedure was performed as per the manufacturer's instruction. Data were analyzed using the supplied software. A 2.5-fold difference of cytokine levels was considered significant. The contents of several selected cytokines were further measured by ELISA (for CNTF and IGF-1) or immunoblotting (PDGF-AA and bFGF).

Phosphorylation of Erk, Akt, CREB, and STAT3 was determined by immunoblotting. Cells were seeded on poly-l-lysine-coated 6-well plates and incubated in PDGF/bFGF-free medium (with insulin) for 48 h, and then changed into insulin-free medium for additional 24 h. Following washing in HBSS, cells were treated with the conditioned medium. At 15, 30, and 120 min, cells were washed twice with ice-cold PBS and detached from the culture surface using a cell scraper. Cells were pelleted by centrifugation at 4°C and lysed in the cell lysis buffer (Millipore) supplemented with protease inhibitor cocktails (Sigma, St. Louis, MO). Cell lysis was then centrifuged at 12,000 rpm for 20 min. The supernatant was collected and total protein contents were determined using BCA method (Pierce, Rockford, IL). Denatured samples were subjected to sodium dodecyl sulfate polyacrylamide gel electrophoresis (SDS-PAGE) and proteins were transferred to nitrocellulose membranes. For immunoblotting, the membranes were first blocked with 5% nonfat milk/1% BSA in PBS for 2 h at RT, and then incubated with primary antibodies overnight at 4°C (pAkt and pERK 1:500, pSTAT3 and pCREB 1:1000). Following washing, membranes were incubated with horseradish peroxidase-conjugated secondary antibody, and signals were detected using the advanced ECL system. The membranes were stripped and reprobed for the total form of Akt, Erk, STAT3, and CREB (1:2000).

### Data analysis

Data for cell survival over multiple time points were analyzed using repeat-measure analysis of variance (ANOVA). All other data were analyzed by one-way ANOVA followed by post hoc Tukey's test. All data were presented as mean ± SEM. A value of *P* < 0.05 was considered statistically significant.

## Results

### ACDM and MCDM protect OPC against growth factor withdrawal-induced degeneration

**Figure 1:**
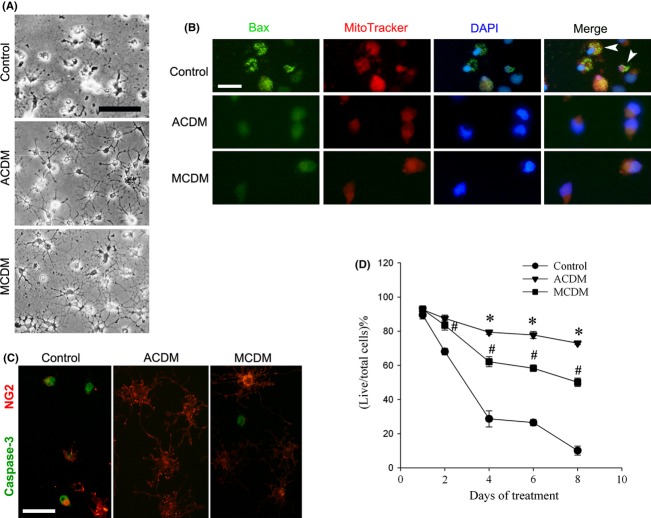
ACDM and MCDM protect OPCs against growth factor withdrawal-induced apoptosis, as well as support long-term OL survival. (A) Representative phase contrast micrographs show that OPCs maintained in the control medium (without growth factors) started to degenerate at 48 h following growth factor withdrawal, while OPCs maintained in ACDM or MCDM remained intact and viable. (B) Triple-labeling of OPCs with Bax, MitoTracker, and DAPI revealed that at 24 h, many cells in the control showed typical Bax translocation from cytosol to mitochondrial membrane (arrowheads), which were not observed in cells cultured in ACDM or MCDM. (C) Numerous OPCs in the control medium showed positive immunostaining of activated caspase-3, while very few, if any, were found in ACDM or MCDM-treated cultures, 24 h following the exposure. (D) Although both ACDM and MCDM could support long-term OL survival, ACDM was significantly more effective than MCDM after 48 h. #*P* < 0.01 versus control; *P < 0.01 versus MCDM. Data are mean ± SEM from three independent experiments. Scale bars: 50 μm.

OPCs require trophic support for their survival, therefore growth factor deprivation can trigger OPC degeneration through apoptosis. To test whether the conditioned medium could prevent OL degeneration, cells were incubated with ACMD, MCDM, or the control medium (without growth factors). Within first few days, a large number of cells degenerated in the control, thus leaving only a small percentage of live cells in a long-term culture. Typically, the degenerative OPCs showed apoptotic characteristics such as shrunk in cell bodies, retraction in the processes, and increase in the brightness under invert microscopy at 48 h (Fig. 1A). Immunocytochemistry data showed that a considerable number of cells in the control were already immunopositive for caspase-3 at 24 h (Fig. 1C), further confirming that cells died via apoptosis. In contrast, both ACDM and MCDM significantly prevented OL degeneration triggered by growth factor withdrawal, which was associated with their ability to suppress Bax translocation from cytosol to mitochondria membrane. As shown in Figure 1B, the punctate colabeling pattern of Bax with MitoTracker was noted in many cells in the control, but very few, if any, were noted in the condition medium-treated cultures, suggesting that the conditioned medium was able to interrupt the intrinsic, caspase-dependent apoptotic pathway.

We then tested whether the condition medium could also support long-term OL survival. As shown in Figure 1D, the survival rate of the control cells declined sharply due to lack of trophic factors, and only 10.1% of cells survived after 8 days of culture. In contrast, there were significantly more survived cells in ACDM- or MCDM-exposed cultures. However, although ACDM and MCDM equally protected cell death in the first 48 h, ACDM was significantly more effective than MCDM in supporting OL survival in the long-term cultures (Fig. 1D). Cell survival rates were 27.8%, 33.4%, and 50% higher in ACDM than in MCDM at 4, 6, and 8 days, respectively.

### ACDM, but not MCDM, promotes OPC proliferation

**Figure 2:**
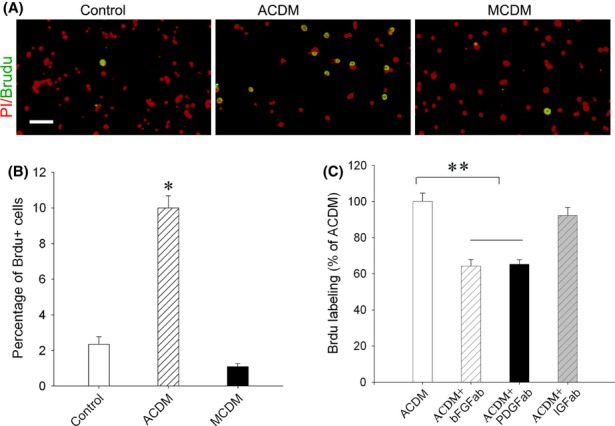
ACDM, but not MCDM, promotes OPC proliferation. After being exposed to the control or the conditioned medium for 48 h, OPC proliferation was assessed by BrdU labeling. (A) Representative photographs show that the number of BrdU+ cells, which was minimal in the control and MCDM group, was significantly higher in ACDM-treated cultures. (B) Quantification of cell proliferation by calculating the percentage of BrdU+ to total cells (PI counterstained nuclei). (C) Blocking PDGFaa and bFGF, but not IGF-1, by their specific neutralizing antibodies, significantly reduced ACDM-enhanced OPC proliferation. *P < 0.01 versus MCDM and the control, and **P < 0.01 versus ACDM. Data are mean ± SEM from three independent tests. Scale bar: 100 μm.

It is possible that the better survival effect of the ACDM on OLs is due to, at least partially, mitotic effect, as growth factor such as PDGF can promote both survival and proliferation of OPCs. To address this issue, we then examined OPC proliferation using BrdU labeling. The percentage of BrdU+ cells was significantly increased in cultures exposed to ACDM (Fig. 2B), but not MCDM (Fig. 2C) or the control (Fig. 2A). To further identify the specific factors that mediate ACDM-enhanced OPC proliferation, the activity of PDGFaa, bFGF, and IGF-1, three major cytokines known to be secreted by astrocytes, were blocked using neutralizing antibodies. The data showed that blocking PDGFaa and bFGF, but not IGF-1, significantly reduced the number of BrdU+ cells in ACDM-exposed cultures (Fig. 2E).

Although ACDM significantly promoted OPC proliferation, the number of BrdU+ cells only accounted for 10% of total cells (Fig. 2B), suggesting that newly generated cells only partially contributed to the higher rate of OL survival noted in ACDM- versus MCDM-treated cultures. By assessing the number of both apoptotic cells (pyknotic nuclei) and total cells (pyknotic nuclei and intact nuclei combined) at 8 days of treatment, it showed that the percentage of degenerated cells was significantly lower in ACDM-treated (22.2%) compared with MCDM-treated cultures (41.9%), while the total number of cells was 11.5% higher in ACDM versus MCDM-treated cultures.

### MCDM, but not ACDM, enhances OL differentiation

**Figure 3:**
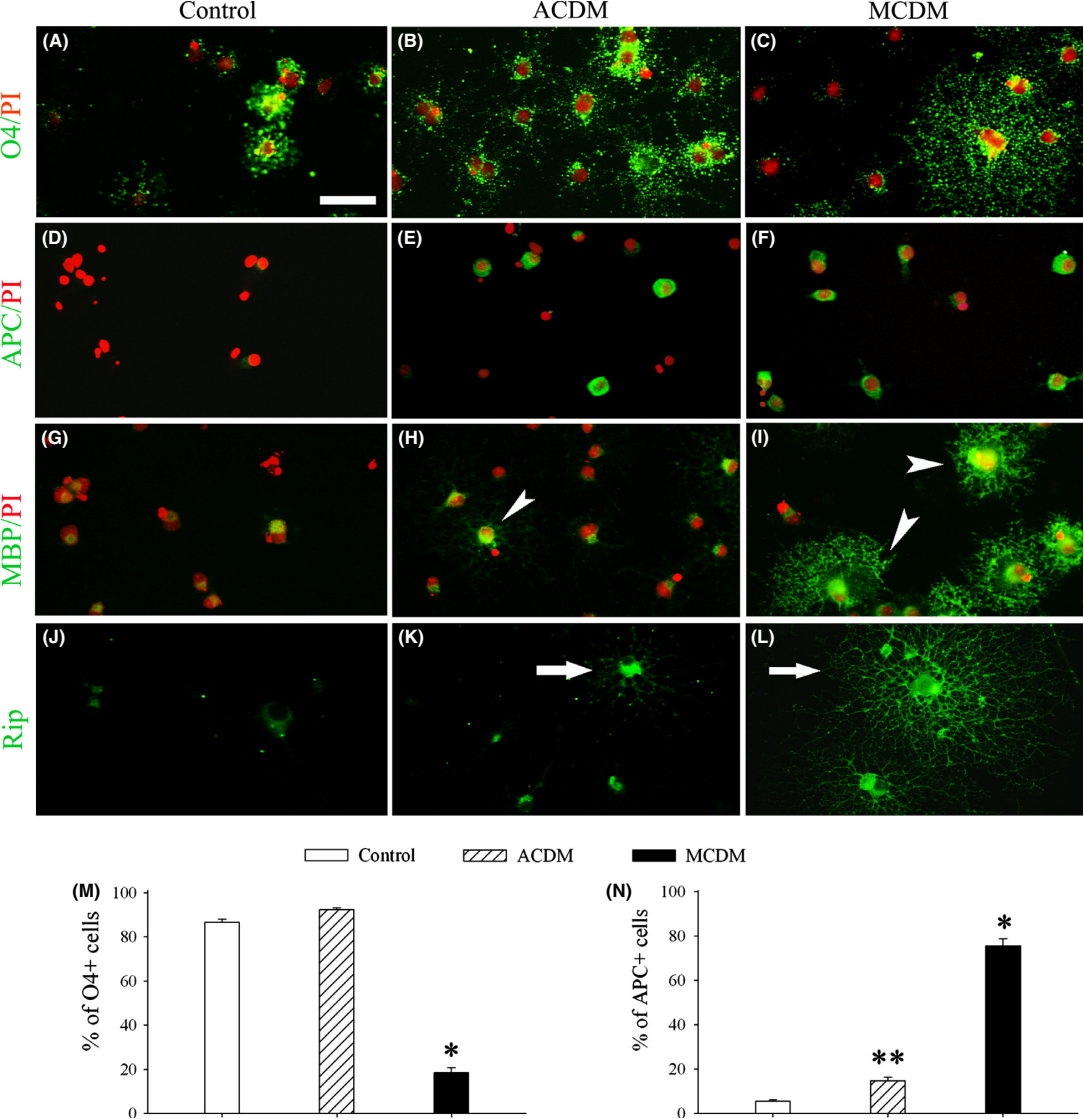
MCDM, but not ACDM, strongly enhances OL differentiation. (A–L) Representative photographs show the effects of ACDM and MCDM on OL differentiation, as assessed using a panel of antibodies against developmental stage-specific OL markers (see text for details), 8 days after the exposure. Most cells in the control medium degenerated as evidenced by pyknotic nuclei (red, PI labeled), while few survival cells remained undifferentiated as indicated by O4+ (A), APC- (D), and MBP- (G) phenotype. Although ACDM greatly enhanced OL survival, it had only weak effect of OL differentiation, indicated by high percentage of O4+ cells (B) and lower percentage of APC+ cells (E), as well as weakly immunostained MBP that was mostly detected in cell somata (an arrowhead in H). In contrast, MCDM-exposed cultures had fewer O4+ (C) but more APC+ cells (F), and the differentiated cells were intensively labeled with MBP in both somata and processes (arrowheads in I). Rip immunostaining revealed fine processes in MCDM-exposed (arrow in L) versus ACDM-exposed (arrow in K) or the control (J) cultures. Scale bar: 50 μm. Quantification of O4+ and APC+ cell number are shown in (M) and (N), respectively. *P < 0.01 versus control or ACDM; **P < 0.05 versus control.

OLs maintained in MCDM displayed profound morphological changes, which were readily discernable under the invert microscopy as early as 24 h. The most notable change was the development of comprehensive process network (Figs. 1A and 3). To better characterize the effects of the conditioned medium on OL differentiation, a panel of OL developmental markers with distinct immunocytochemical labeling patterns was used (O4 and MBP label both somata and processes of immature OLs and mature OLs, respectively; APC labels only somata of mature OLs; and Rip labels fine processes of both immature and mature OLs). As shown in Figure 3, MCDM strongly accelerated OL differentiation as evidenced by higher percentages of APC+ mature OLs (Fig. 3F) with concomitant lower percentages of O4+ OPCs (Fig. 3C) in 8-day cultures. In contrast, although ACDM also significantly increased APC+ mature OLs (Fig. 3E) when compared to the control, the effect was much weaker than that of MCDM (14.7 ± 1.6 vs. 75.6 ± 3.1, *P* < 0.01). In fact, cells maintained in ACDM remained primarily as undifferentiated O4+ immature OLs (Fig. 3B and M) with only a slight (but significant) increase in the percentage of APC+ cells (Fig. 3E and N), suggesting a limited effect on OL differentiation by ACDM. Consistently, the effect of the conditioned medium on OL differentiation could be clearly visualized by morphological changes. Intensive MBP (Fig. 3I) and Rip (Fig. 3L) immunoreactivity, especially in cell processes (Fig. 3I), was observed in MCDM-treated OLs, whereas it was rather weak in ACDM-treated (Fig. 3H and K, respectively) or the control cells (Fig. 3G and J, respectively). Most notably, the primary, secondary, and tertiary branches of OL processes, as well as the process network, were clearly visible with Rip immunostaining in MCDM-treated (Fig. 3L), but not ACDM-treated (Fig. 3K) or the control cultures (Fig. 3J).

### Patterns of cytokine in MCDM and ACDM are different as revealed by protein assay

**Figure 4:**
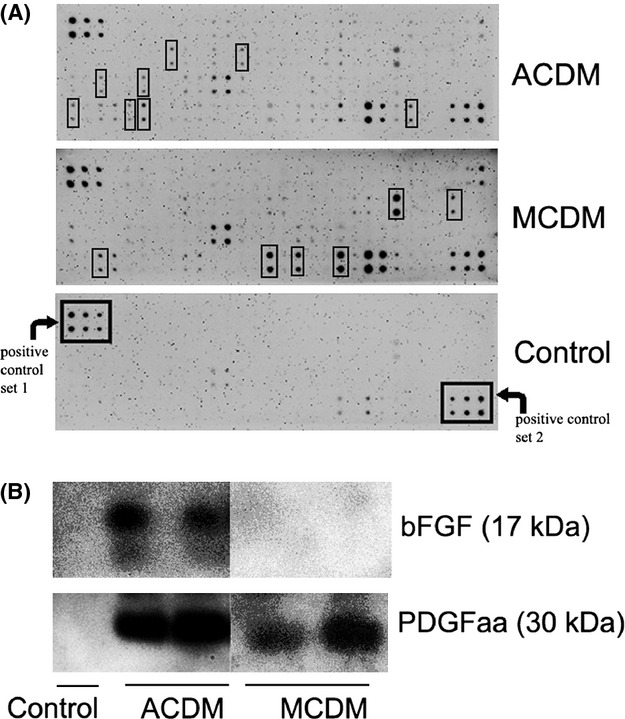
Cytokine array reveals distinct cytokine patterns in ACDM and MCDM. (A) A protein array was used to profile 90 cytokines in ACDM and MCDM. Each cytokine was detected in duplicate. Differentially expressed cytokines (defined as >2.5-fold higher) are highlighted with black boxes (see text for details). (B) PDGFaa and bFGF were further detected in the conditioned medium by Western blot, which showed that ACDM contained higher levels of both growth factors than MCDM.

Next, we used a protein array to define the patterns of cytokine in the conditioned medium (Fig. 4A). Among the 90 cytokines, seven were detected at higher levels in ACDM than in MCDM, including CNTF, bFGF, bFGF-binding protein, PDGF-AA, growth hormone, TIMP-1, and thrombospondin. In contrast, levels of E-selectin, fractalkine (also known as CX3CL1), neuropilin-2, IL-2, IL-5, and vascular endothelial growth factor (VEGF) were significantly higher in MCDM than in ACDM. To validate the protein array data, as well as to compare the levels of cytokines that are not included in the array but are known to be important for OL development in vivo, several selected cytokines were further measured quantitatively by ELISA or Western blot. The ELISA data revealed a striking difference of CNTF and IGF-1 levels between ACDM and MCDM. CNTF concentration was 12.5-fold higher in ACDM than in MCDM (642.7 ± 6.1 pg/mL vs. 51.5 + 1.5 pg/mL, *n* = 3), while the levels of IGF-1 was 6.3-fold higher in MCDM than in ACDM (424.7 ± 57.6 pg/mL vs. 66.7 ± 4.0 pg/mL, *n* = 3). Consistent with the cytokine array data, Western blot data showed similar patterns of PDGF and bFGF in the conditioned medium. Strong bFGF bands were only detected in ACDM, whereas PDGFaa bands were detected in both medium; however, its levels were 2.5-fold higher in ACDM than in MCDM (Fig. 4B).

### ACDM simultaneously activates multiple signaling pathways linked with cell survival and growth, while MCDM only weakly activates CREB pathway

**Figure 5:**
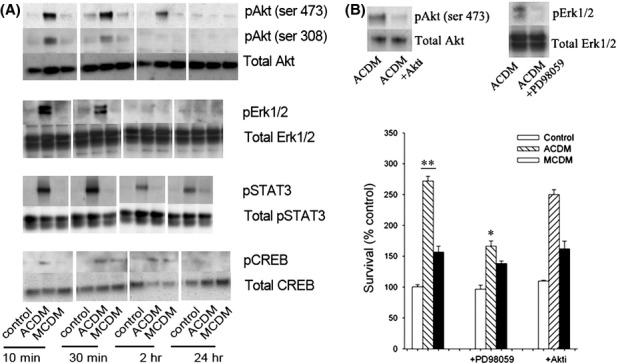
Activation of multiple signaling pathways in OPCs upon exposure to the conditioned medium. (A) OPCs were exposed to the control or conditioned medium for indicated times and the phosphorylated as well as the total form of several signaling pathways were detected by Western blot. All pathways were rapidly and strongly activated in OPCs by ACDM, while only a weak, but delayed activation of CREB was detected in OPCs by MCDM. (B) Pretreatment of OPCs with PD98059, which specifically inhibited ACDM-induced Erk activation (upper panel), abolished ACDM-mediated OL survival. In contrast, pretreatment with Akt inhibitor showed no effect. **P < 0.01 versus control; *P < 0.01 versus ACDM.

The preferential effect of ACDM on OL proliferation versus MCDM on OL differentiation is likely due to differentially activated intracellular signaling pathways. To test this idea, several putative signaling pathways that are frequently linked to OL proliferation, survival, and myelination were assessed in OPCs upon exposure to the conditioned medium. The phosphorylated Akt (both ser308 and ser473), Erk1/2, CREB, and STAT-3, as well as the total form of these kinases, were detected by Western blot following treatment with the conditioned medium (Fig. 5A). Surprisingly, ACDM strongly activated all these pathways in OPCs, in a time-dependent manner. pAkt, pErk, and pCREB were rapidly activated in OPCs but then quickly returned to baseline level within 2 h, while pSTAT3 levels remained high at 24 h. In contrast to the simultaneous activation of multiple signaling pathways by ACDM, MCDM only weakly activated CREB pathways in OPCs. Using specific inhibitors, we further showed that PD98059 (Erk1/2 inhibitor), but not SH5 (Akt inhibitor), significantly reduced ACDM-enhanced OL survival (Fig. 5B).

### MCDM, but not ACDM, strongly promotes myelination in vitro

**Figure 6:**
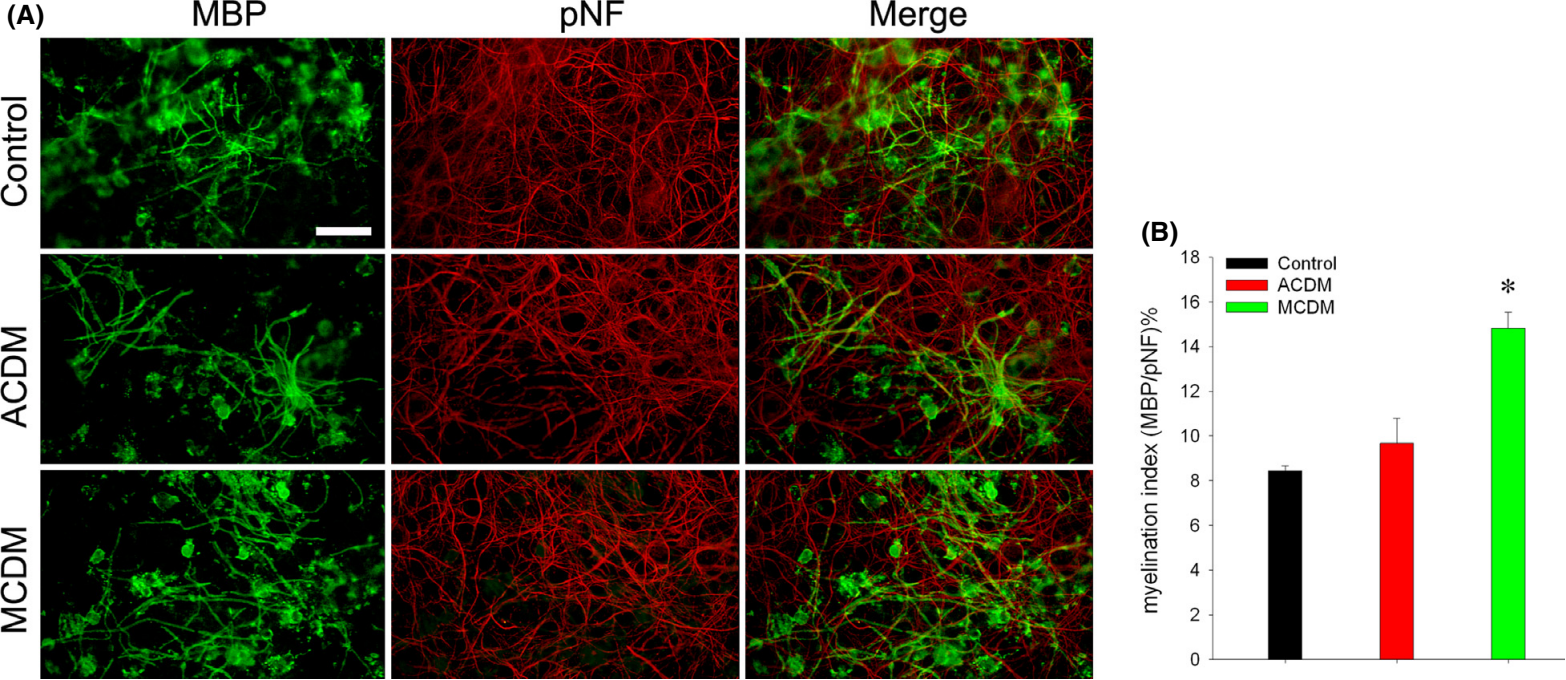
MCDM enhances myelination in vitro. Myelinating OL-neuron cocultures were exposed to ACDM, MCDM, or the control medium from DIV7 to DIV35. Myelinated internodes were revealed by coimmunolabeling with MBP and pNF (merged panels in A), and myelination index was assessed by calculating the percentage of areas occupied by MBP+/pNF+ myelinated versus total pNF+ axons (B). The myelination index was significantly higher in MCDM-treated, but not ACDM-treated cultures, compared with the control. *P < 0.01 versus control or ACDM. Scale bar: 100 μm.

Finally, the effect of MCDM and ACDM on myelination was tested in a myelinating neuron-OL coculture. After being exposed to ACDM or MCDM for 4 weeks, the number of myelinated internodes (MBP/pNF double-labeled axons) was determined. Consistent with its ability to enhance OL differentiation, MCDM exposure significantly increased the extent of myelination (Fig. 6A and B). In contrast, ACDM had no discernable effect on myelination. It appears that neither conditioned medium affected axonal density (Fig. 6A, pNF panels).

## Discussion

The major finding of the current study is that ACDM and MCDM preferentially affect OL development and myelination, which are underlined by distinct cytokine patterns in the conditioned medium and by selectively activated intracellular signaling pathways in OLs.

The finding that ACDM promotes OPC proliferation is consistent with early studies. For example, OPCs grown on top of astrocyte monolayer had higher proliferation rate than without astrocytes, whereas PDGF was identified as the primary mitogen (Hardy and Reynolds 1991). Based on in vivo studies, PDGF has been suggested as one of the key growth factors to regulate OPC numbers during early OL development. Because PDGF is both a mitogen and a survival factor, competing for PDGF appears to be a fundamental mechanism to control the number of OPCs needed to differentiate into mature OLs and to myelinate axons (Barres et al. 1992, 1994). Similar to PDGF, bFGF is another key mitogen for OPCs. Importantly, bFGF synergizes with PDGF to promote OPC proliferation and survival (Grinspan 2002). Therefore, it is likely that higher levels of PDGF and bFGF in ACDM were responsible for its potent survival and mitotic effects on OPCs.

The selective effects of ACDM and MCDM on OL developmental phenotypes are in general accordance with cytokine array data which showed distinct cytokine patterns in ACDM and MCDM. Most factors detected at higher levels in ACDM are known to play mitotic and/or survival roles in OL development. It is worth noting that PDGF can work together with bFGF to promote OPC proliferation and survival, and such ability is inversely linked to their strong inhibition for OL differentiation (Tang et al. 2000). Interestingly, TIMP-1, which was also detected at higher levels in ACDM, has recently been shown to regulate the number of NG2+ OPCs in the developing mice brain (Moore et al. 2011). One exception is CNTF. Although CNTF is a potent trophic factor for OPCs, it also strongly promotes OL differentiation (Louis et al. 1993; Mayer et al. 1994). The fact that CNTF levels are at high levels raises the question as why only a weak effect of ACDM on OL differentiation was observed? One of the possible explanations is that CNTF-activated signaling pathway(s) pertinent to OL differentiation was masked by proliferating/survival pathways activated by other cytokines (such as PDGF and bFGF), for example, due to cross talks between these intracellular signaling pathways. In fact, multiple signaling pathways including Akt, Erk, STAT3, and CREB were activated in OLs following ACDM exposure, while these pathways are frequently linked to cell survival and proliferation. As for those factors at higher levels in MCDM, IGF-1 is one of the best characterized trophic factors for OL development and myelination, both in vivo and in vitro. For example, mice with IGF-1 overexpression exhibit increased number of OLs and enhanced myelination in the brain (D'Ercole et al. 2002). In vitro, IGF-1 not only provides strong trophic support (Cui et al. 2005) but also facilitates OPC differentiation (Pang et al. 2007). Less known is the effect of VEGF on OLs and/or myelination, but several studies suggest that VEGF is involved in OPC migration but not proliferation (Hayakawa et al. 2011, 2012). Finally, other factors, including E-selectin, CX3CL1, IL-2, IL-5, are either cytokines or chemokines. While effects of these individual cytokine/chemokines on OL development are less clear, recent studies suggest that many cytokines and chemokines play roles in early neural/glia development. For example, it has been suggested that CX3CL1 may mediate microglia–neuron communication and synaptogenesis (Hoshiko et al. 2012), and several chemokines have recently been identified as potential regulators for OL development and myelination (Patel et al. 2010).

The underlying mechanisms for OL differentiation are rather complex. Many cytokines, including members of IGF family, CNTF/LIF (leukemia inhibitory factor) family of cytokines, certain chemokines and neurotrophins, have been shown to enhance OL differentiation (Mayer et al. 1994; Heinrich et al. 1999; Hsieh et al. 2004; Xiao et al. 2009). Those extracellular ligands activate intracellular signaling pathways leading to transcriptional regulation of myelin proteins such as MBP, PLP, and CNPase (Wegner 2008; Emery 2010b). Although the current work did not explore much of the mechanisms underlying MCDM-enhanced OL differentiation, we speculate that IGF-1 may play such a role, as IGF-1 levels were more than sixfold higher in MCDM than in ACDM. Many studies have reported that Akt and Erk pathways are involved in OL differentiation (Pang et al. 2007; Guardiola-Diaz et al. 2012). However, in the current study neither pAkt nor pErk was detected in OLs upon MCDM exposure. Interestingly, CREB was weakly activated in OLs, suggesting a possible involvement of this signaling pathway in MCDM-enhanced OL differentiation. In fact, accumulating evidence suggests that CREB is a potential downstream pathway involved in OL differentiation (Paez et al. 2004; Shiga et al. 2005; Bhat et al. 2007). Whether activation of CREB pathway principally underlies MCDM-enhanced OL differentiation needs to be addressed in future studies.

Myelination is a relatively late developmental event of mammalian nervous system, which is fine turned by a comprehensive transcription network (Bradl and Lassmann 2010). Neuron-derived factors, including neurotrophins and axonal surface molecules are known to play critical roles in initiating myelination (Xiao et al. 2009). As for glial cells, astrocytes have been implicated to affect myelination, presumably through secreted factors. However, most of the data regarding the role of astrocytes in myelination are observed from studies of multiple sclerosis, in which astrocytes are often activated. Nevertheless, a few studies directly investigated the effect of astrocytes on myelination in cell cultures. For example, Ishibashi et al. (2006) reported that astrocytes could promote myelination in response to neuronal activity by releasing of LIF. Watkins et al. (2008) showed that the presence of astroctyes in myelinating cultures could increase the speed of myelin wrapping around axons, but was not required for the initiation of myelination. The lack of myelin promoting effect of ACDM in the current study is not clear, but may be due to several factors. First, astrocytes were presented in the cocultures (Pang et al. 2012), and astrocyte-derived factors may contribute to the baseline level of myelination. Therefore, ACDM may no longer show additional effect. Second, the phenotypes of astrocytes seem to be important in determining whether astrocytes affect myelination positively or negatively (Nash et al. 2011). In the current study, we subcultured the primary cells in order to obtain astrocytes at high purity, thus, this may potentially change their phenotypes.

At present, the effect of microglia on myelination is largely unknown. Considering that microglia can enhance OL differentiation and support cell survival (Pang et al. 2000, 2010; Nicholas et al. 2001), it is readily to postulate that microglia are beneficial to myelination, although direct evidence is lacking. To the best of our knowledge, the current study is the very first to demonstrate that MCDM could enhance in vitro myelination. Our previous study has shown that, similar to in vivo, compact myelin sheath and nodal structures were formed around axons in the coculture system, as examined by electron microscopy (Pang et al. 2012), suggesting that the increased number of myelinated internodes are comparable to their in vivo counterparts. However, it is premature to conclude that microglia play a similar role in myelination in development, and/or myelin repair in certain CNS disorders.

In summary, our present data reveal distinct effects of ACDM and MCDM on OL development and myelination in vitro. These findings may have both physiological and pathological implications. As for the later, compromising of OL development and myelination are core features in certain neurological disorders such as white matter damage in premature infants and multiple sclerosis in adults. Therefore, understanding the basic mechanisms by which astrocyte and microglia regulate normal OL development and myelination are essential to elucidate their pathological roles and will help to identify molecules/pathways for future intervention.
